# Treprostinil Reconstitutes Mitochondrial Organisation and Structure in Idiopathic Pulmonary Fibrosis Cells

**DOI:** 10.3390/ijms241512148

**Published:** 2023-07-29

**Authors:** Lei Fang, Wei-Chih Chen, Peter Jaksch, Antonio Molino, Alessandro Saglia, Michael Roth, Christopher Lambers

**Affiliations:** 1 Pulmonary Cell Research, Department Biomedicine & Clinic of Pneumology, University & University Hospital Basel, CH-4031 Basel, Switzerland; fanglei.83@icloud.com (L.F.); michael.roth@usb.ch (M.R.); 2 Department of Chest Medicine, Taipei Veterans General Hospital, Taipei 11217, Taiwan; wiji.chen@gmail.com; 3 Institute of Emergency and Critical Care Medicine, National Yang Ming Chiao Tung University, Taipei 11266, Taiwan; 4 Thoracic Surgery, University Hospital Vienna, Währinger Gürtel 10-14, 1090 Vienna, Austria; peter.jaksch@meduniwien.ac.at; 5 Department of Respiratory Diseases, University of Naples, Federico II, via S. Pansini 10, 80131 Naples, Italy; molinotonio@libero.it; 6 Department of Respiratory Diseases, AO dei Colli, via L. Bianchi snc, 80131 Naples, Italy; alessandro.saglia@ospedalideicolli.it; 7 Department of Pneumology, Ordensklinikum Linz/Elisabethinen, Fadingerstr. 1, 4020 Linz, Austria

**Keywords:** idiopathic pulmonary fibrosis, mitochondrial dysfunction, treprostinil, cellular signalling, remodelling

## Abstract

Idiopathic pulmonary fibrosis (IPF) presents as an incurable change in the lung tissue and mitochondrial dysfunction of unknown origin. Treprostinil, a prostacyclin analogue, has been suggested for IPF therapy. This study assessed the effect of treprostinil on the cAMP signalling and mitochondrial activity in healthy lung fibroblasts and fibroblast-like cells from IPF patients. Six control fibroblast strains and six fibroblast-like IPF cell strains were isolated and expanded from freshly resected lung tissue. The cells were grown to confluence before being treated with either transforming growth factor (TGF)-β1, treprostinil, their combination, or a vehicle for up to 2 days. Mitochondria-regulating proteins were analysed using Western blotting and immunofluorescence, and the mitochondria were analysed using cytochrome C, mitochondrial cytochrome C oxidase II (MTCO2), and MTCO4. The IPF cells showed an increased rate of damaged mitochondria, which were significantly reduced when the cells were treated with treprostinil over 24 h. In the control cells, treprostinil prevented TGF-β-induced mitochondrial damage. Treatment with treprostinil modified the expression of several mitochondria-regulating proteins. In both cell types, treprostinil upregulated the expression of PTEN, p21(Waf1/Cip1), beclin1, LC3 II, parkin, PINK1, MTCO2, and MTCO4. In contrast, treprostinil downregulated the phosphorylation of mTOR and the expression of p62, mitofusin1, and mtiofusin2 in IPF cells. This might explain the reduced mitochondrial damage observed in treprostinil-treated IPF cells and suggest an improvement in the mitochondrial function in IPF. In this study, treprostinil improved mitochondrial impairment in vitro, which might, in part, explain the beneficial clinical effects documented in patients.

## 1. Introduction

Idiopathic pulmonary fibrosis (IPF) affects 3 million people worldwide and is a progressive condition of irreversibly declining lung function, which is assumed to result from a wide range of structural changes to lung tissues [1]. An epidemiological study suggested a worldwide increase in IPF, which could be due to either improved diagnoses or a real increase in the incidences in the aging population [2].

The cause of IPF remains elusive. The pathological features include the aging of alveolar epithelial cells, the replacement of epithelial cells by fibroblasts, and mitochondrial dysfunction [3,4,5]. Dysfunctional mitochondria have been described in different cell types that are relevant to the pathogenesis of IPF and that affect the balance of epithelial cells and fibroblasts [6]. Mitochondrial damage occurs mainly in the fibroblasts/myo-fibroblasts of IPF patients, and is characterized by a reduced mitochondrial mass and the malfunction of the mitochondrial membrane caused by oxidative stress [3]. These cellular pathologies of IPF might be linked to faster aging and telomere shortening [7]. Mitochondrial malfunction might also result from the interaction between mitochondria and the extracellular matrix (ECM), which is modified in IPF [8]. This complex network between the ECM, immunity, and lipid metabolism is reported in IPF patients at an early stage [9].

Mitochondria control energy consumption, protein synthesis, the production of radical oxygen species (ROS), and mitophagy [10]. Somatic mutations in mitochondrial DNA and reduced levels of cytochrome C oxidase subunit II (MTCO2) occur more frequently in the tissues from lung fibrosis patients [5]. Damaged mitochondria have been reported in the structural cells of tissues and lung-infiltrating macrophages in IPF patients [6,11,12]. High levels of damaged mitochondrial DNA predict the death of IPF patients [13].

The mitochondrial structure changes between fusion and fission, which affects the function of mitochondria [14]. The loss of mitochondrial integrity plays an essential role in the pathogenesis of lung fibrosis and premature aging [3,15]. This hypothesis is supported by the observation that autophagy- and mitophagy-regulating proteins are reduced in the bronchial alveolar lavage fluid (BALF) of IPF patients [12]. Mitochondrial function and the life span of healthy human fibroblasts are regulated by the mammalian target of rapamycin (mTOR) [16]. In addition, deregulated fibroblast differentiation and reduced mitophagy is linked to mTOR signalling in lung fibrosis, and is modified by pirfenidone [17]. In a placebo-controlled phase-III trial, inhaled treprostinil (a prostacyclin analogue) improved the forced vital capacity (FVC) compared to a placebo within 16 weeks in 326 patients with interstitial lung diseases [18].

In pulmonary fibrosis, the reprogramming of cell function was linked to damaged mitochondria, the deregulated activation of mitogen-activated protein kinases (MAPKs) and peroxisome proliferator-activated receptor gamma coactivator 1α (PGC-1α), and increased mitochondrial reactive oxygen species (ROS) synthesis [4]. In other conditions, cyclic adenosine monophosphate (cAMP) activation was followed by protein kinase B (Akt) phosphorylation, mTOR signalling, and p70S6 kinase (p70S6K) and PGC-1α activation, leading to mitochondrial fission [19]. It would be of interest if cAMP activation by treprostinil affects this signalling pathway and, therefore, mitochondrial activity.

In vitro, treprostinil prevented fibroblast proliferation by activating cAMP [20,21,22]. This action may occur through the inhibition of the pro-inflammatory/pro-proliferative signalling cascade of mTOR by cAMP, phosphatase, and the tensin homologue (PTEN), as described in airway smooth muscle cells [23]. The loss of PTEN contributes to the transformation of fibroblasts in IPF [24] and is correlated with the severity of the disease [25].

Despite extensive research, therapeutic options for the treatment of IPF are limited, and research investigating additional compounds is ongoing. Nathan et al. demonstrated the beneficial and clinically relevant effect of treprostinil in patients with IPF and pulmonary hypertension, suggesting that prostacyclins might exert an inimitable inhibitory effect on fibrosis beside its potency on vasodilation. However, there is no evidence as to whether the newly identified mechanism of mitochondrial damage leading to fibrosis is affected by prostacyclins such as treprostinil.

This study assessed the mechanism controlling mitochondrial fusion and fission in IPF-derived fibroblast-like cells.

## 2. Results

### 2.1. Mitochondrial Damage in IPF Cells

**Figure 1:**
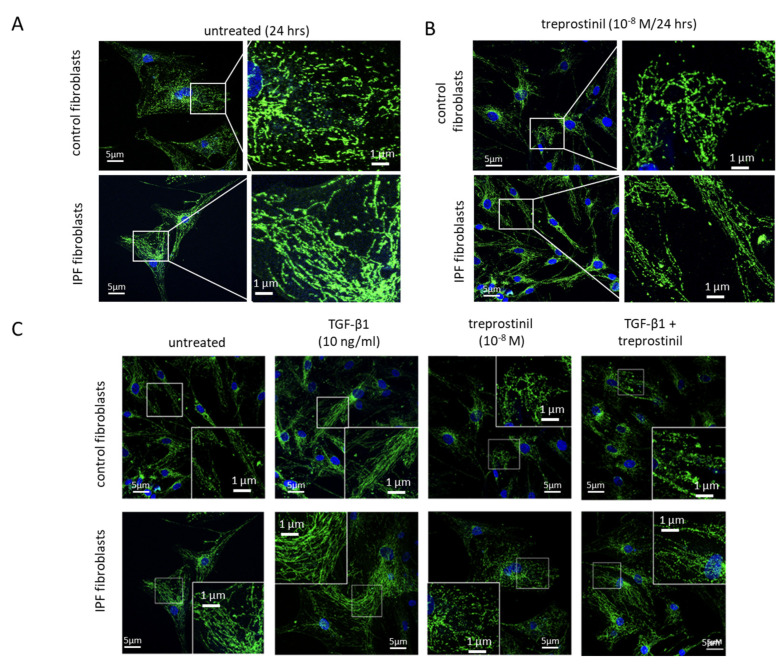
Mitochondrial fission is reduced by treprostinil. Representative immunofluorescence photography of mitochondrial protein expression and morphology, as determined by cytochrome C staining, in (**A**) control cells and (**B**) IPF cells. (**C**) The effect of treprostinil (10*^−^*^8^ M) on TGF-β1 (5 ng/mL/24 h)-induced mitochondrial protein expression and fusion in control and IPF cells. The indicated areas were enlarged for better visibility of mitochondrial structures. Mitochondria: green, nuclei blue by DAPI.

Compared to control fibroblasts, IPF cells contain an increased mitochondrial protein expression and show enhanced mitochondrial fusion, as determined by staining for cytochrome C (Figure 1A). Treatment with treprostinil (10*^−^*^8^ M) over 24 h reduced the mitochondrial protein expression and fusion in both control and IPF cells (Figure 1B). TGF-β1 plays a major role in the pathogenesis of IPF; therefore, both cell types were treated with human recombinant TGF-β1 (5 ng/mL) for 24 h, which significantly increased hypertrophy and the fusion of mitochondria in control and IPF cells (Figure 1C). A pretreatment (30 min) with treprostinil (10^−8^ M) prevented all the effects of TGF-β1 on mitochondria (Figure 1C).

### 2.2. Treprostinil Modulates Mitochondrial Activity and Autophagy

**Figure 2:**
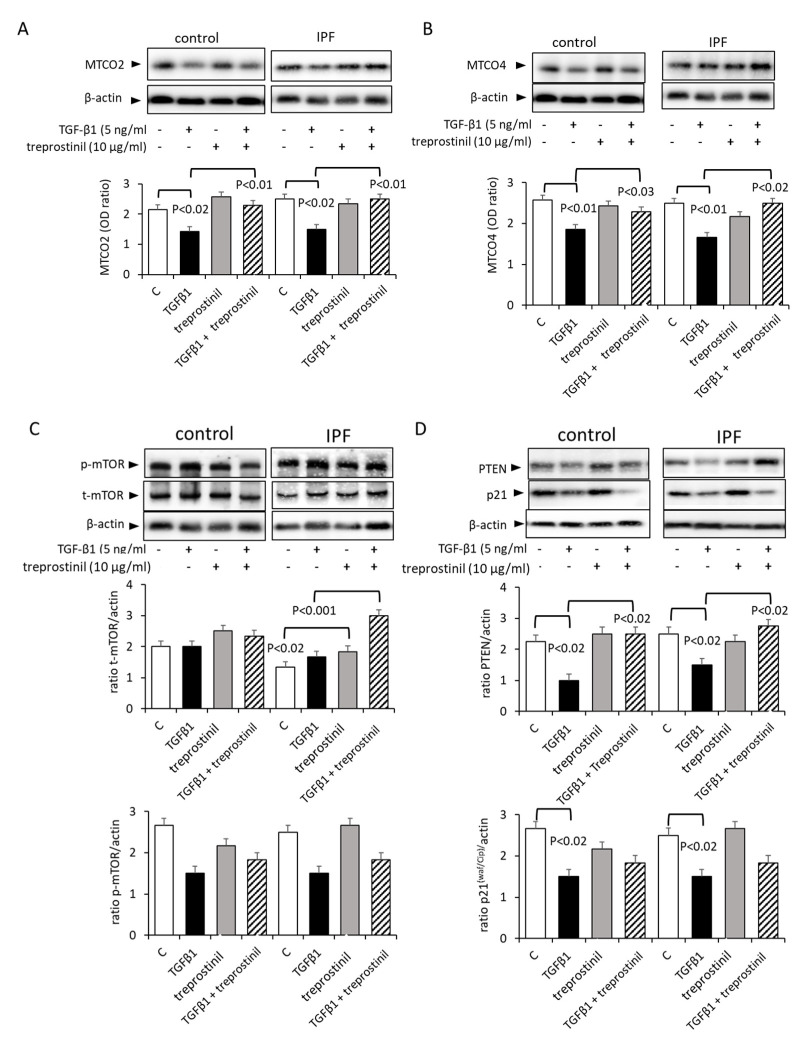
Treprostinil modulates the activation of mitochondria. (**A**) MTCO2-representative Western blot and bar chart analysis of the optical density of six control and six IPF cell strains. (**B**) MTCO4-representative Western blot and bar chart show the means ± S.E.M. of optical density analysis (*n* = 6 for control, *n* = 6 for IPF). (**C**) Treprostinil-modified mTOR phosphorylation (p-mTOR). (**D**) PTEN and p21^(Waf1/Cip1)^ (p21) expression. Similar results were obtained in 5 control and 5 IPF cell strains. β-actin and GPADH served as house-keeping proteins. T-mTOR: total mTOR, C: untreated control cells.

The basal expression of MTCO2 was similar in IPF and control cells (both *n* = 6) (Figure 2A). Treatment with TGF-β1 significantly reduced the expression of MTCO2 in both control and IPF cells (Figure 2A). Treprostinil had no effect on the baseline expression of MTCO2 in both IPF and control cells, but it significantly counteracted the effect of TGF-β1 (Figure 2A).

The baseline expression of MTCO4 was similar in IPF and control cells (both *n* = 6), and this was significantly reduced by the TGF-β1 treatment (Figure 2B). Treprostinil alone had no effect on the baseline expression of MTCO4 in control cells, but reversed the reducing effect of TGF-β1 on the MTCO4 expression (Figure 2B). MTCO2 is down-stream of mTOR and was downregulated by TGF-β1; this effect was counteracted by treprostinil in IPF cells (Figure 2C). In line with this observation, the mTOR inhibitor PTEN [23] was upregulated by treprostinil (Figure 2D). Similarly, the expression of the negative proliferation controller p21^(Waf1/Cip1)^ was upregulated by treprostinil (Figure 2D), confirming the earlier presented data of the drug and overriding the pro-fibrotic action of TGF-β1 [21].

### 2.3. Treprostinil Affects Mitophagy- and Autophagy-Regulating Protein Expression

**Figure 3:**
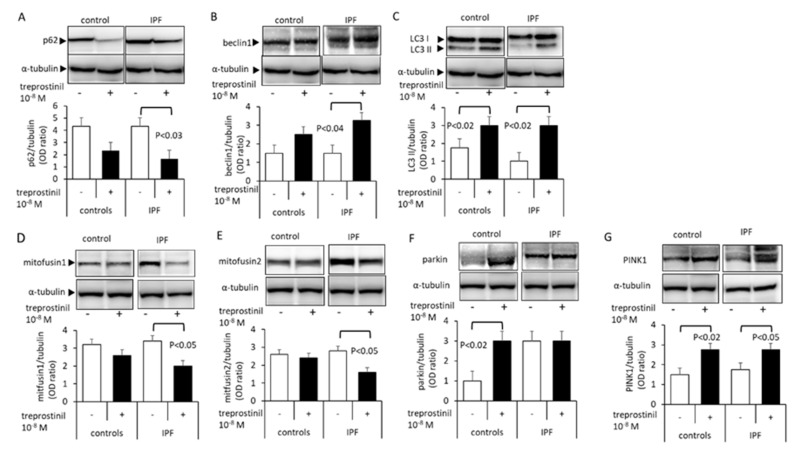
Representative Western blots and OD bar chart analysis for the 24 h effect of treprostinil (blotted as “+”) on mitochondrial regulating proteins: (**A**) p62, (**B**) beclin1, (**C**) LC3 II, (**D**) mitofusin1, (**E**) mitofusin2, (**F**) parkin, and (**G**) PINK1. Bars represent means ± S.E.M. of at least three independent Western blots. The *p*-values were calculated by a paired Student’s *t*-test.

The expression of p62, a protein that regulates protein degradation through autophagy, was downregulated by treprostinil in both control and IPF cells, but this effect achieved significance only in IPF cells (Figure 3A). The basal level of beclin1 was higher in IPF cells compared to controls (Figure 3B). The beclin1 expression in IPF cells was significantly further increased in IPF cells (Figure 3B). LC3 I was more strongly expressed than LC3 II in both cell types and was not affected by treprostinil (Figure 3C). However, LC3 II was significantly increased by treprostinil in both cell types (Figure 3C). Mitofusin1 was significantly reduced by treprostinil in IPF cells, but not in control fibroblasts (Figure 3D). Similarly, the mitofusin2 expression was significantly reduced by treprostinil in IPF cells only (Figure 3E). Parkin was significantly increased by treprostinil, but only in control cells (Figure 3F). In contrast, the expression of PINK1 was significantly increased by the treprostinil treatment in both the control and IPF cells (Figure 3G).

### 2.4. Treprostinil Induces cAMP–Akt–CREB Signalling

**Figure 4:**
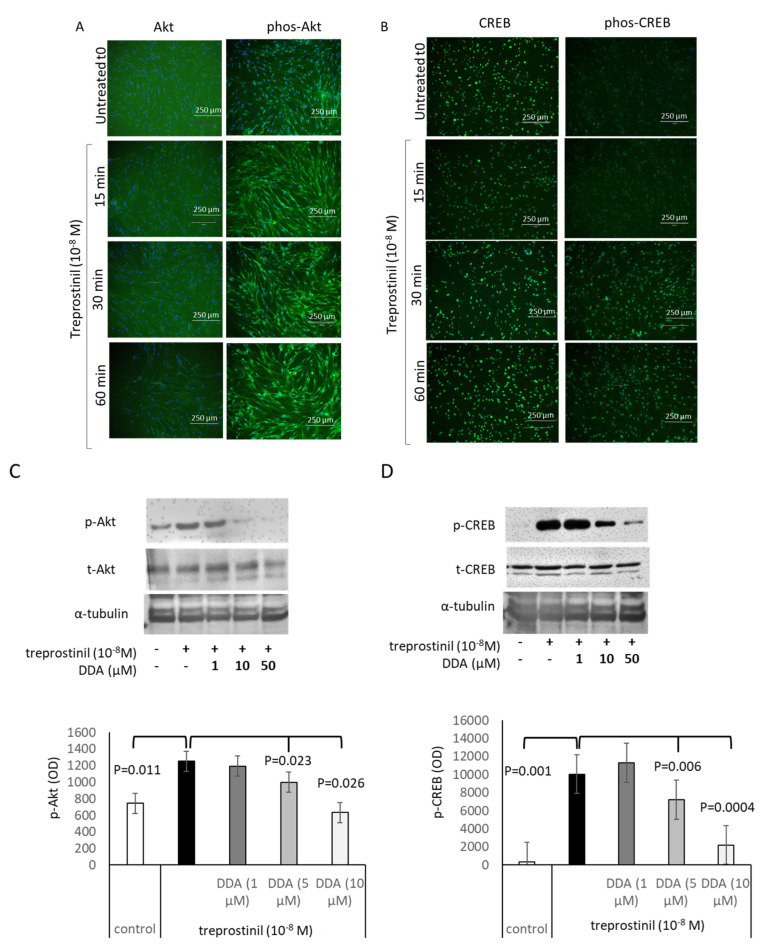
Treprostinil stimulates Akt–CREB signalling in IPF cells. Representative immunofluorescence microscopy for the action of treprostinil (10*^−^*^8^ M) on the expression of (**A**) total Akt (t-Akt), phosphorylated Akt (p-Akt), and (**B**) total CREB (t-CREB) and phosphorylated CREB (p-CREB). Nuclei were stained by DAPI (blue). Similar results were achieved in two additional IPF cell lines. (**C**) Representative Western blots for the concentration-dependent inhibitory effect of DDA (pre-treatment for 30 min.) on treprostinil (10*^−^*^8^ M)-induced expression and phosphorylation of t-Akt and p-Akt and (**D**) t-CREB and p-CREB phosphorylation. Bars show the means ± S.E.M. of the ratio for p-Akt/t-Akt and for p-CREB/t-CREB. The *p*-values (Student’s *t*-test, paired, two-tailed) were calculated from three independent experiments in three IPF cell lines.

As shown by immunofluorescence, treprostinil upregulated the phosphorylation of Akt in the nucleus within 15 min in IPF cells; however, it had no significant effect on the cytosolic Akt expression. Akt phosphorylation remained active for 60 min and declined thereafter (Figure 4A). CREB phosphorylation was upregulated by treprostinil within 30 min and remained active for 60 min (Figure 4B). No activation was observed for Erk1/2 MAPK or p38 MAPK (data not shown). A pre-incubation with 2′,3′-dideoxyadenosine (DDA, 10 μM, 30 min), a specific adenylyl cyclase inhibitor, attenuated the stimulatory effect of reprostinil on Akt (Figure 4C) and CREB (Figure 4D) phosphorylation at the corresponding time point of maximal activation.

## 3. Discussion

**Figure 5:**
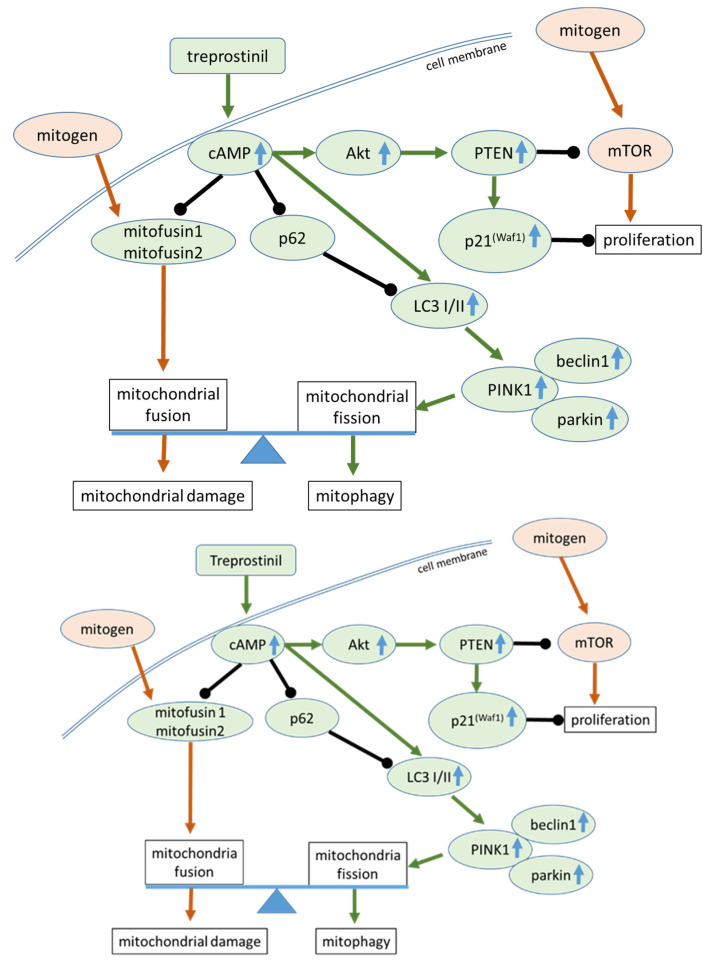
Graphic abstract of the proposed beneficial mitochondria-regulating effect of treprostinil in IPF cells. The upregulation of protein expression is indicated by blue arrows, black bars indicate inhibitory effects, and green and brown arrows indicate stimulatory effects.

Mitochondrial dysfunction is a characteristic of IPF, and has been insufficiently recognized as a therapeutic target [3]. Inhaled treprostinil showed beneficial effects in patients with interstitial lung disease and pulmonary hypertension [18]. Mitochondrial dysfunction has been reported in IPF and is linked to tissue degradation and fibrosis [1,6]. In this study, we investigated the effect of treprostinil, a prostacyclin analogue, on the mitochondrial function and structure in healthy fibroblasts and IPF-lung-derived cells. The presented data support the idea that treprostinil achieves its beneficial effects in IPF patients by reducing the mitochondrial mass and fusion, as summarized in Figure 5.

In an earlier study, we reported that treprostinil activates the dual specificity of phosphatase 1 (DUSP1), and thereby inhibits the mitogenic effect of Erk1/2 MAPK [26]. In the same publication, it was shown that microRNA-21-5p is constitutively over-expressed in IPF cells. In keloid fibroblasts, the inhibition of microRNA-21-5p reduces mitochondria-regulated apoptosis [27]. In glioma cells, cAMP plays an essential role in the regulation of mitochondrial activity and cell apoptosis [28]. The increased resistance to apoptosis is a characteristic of IPF cells linked to mitochondrial dysfunction and fission [29,30]. Furthermore, IPF-derived fibroblasts are insensitive to the apoptosis induced by collagen type-I due to reduced autophagy [31]. In line with these findings, treprostinil reduced collagen type I synthesis while increasing that of fibronectin via cAMP, and thereby reduced inflammation in IPF fibroblasts. Therefore, treprostinil might re-establish the sensitivity of IPF cells to apoptosis [21,22]. Furthermore, we reported earlier that the treatment of IPF cells with treprostinil significantly reduced the expression of the myo-fibroblast marker α-smooth muscle actin [22].

One regulator of autophagy is beclin1, which was downregulated by TGF-β1 and is regarded as a major contributor to the pathogenesis of IPF [32]. In contrast to our data, cAMP and Akt downregulated beclin1 in a mouse model [33]. Another fibrosis model suggested that the epithelial–mesenchymal transition is upregulated by beclin1 [34]. In this context, it should be noted that the function of beclin1 depends on complex formations with other autophagy-regulating proteins such as B-cell lymphoma 2 (BCL2) [35]. This finding is in line with the observation that sustained cAMP signalling is needed for the anti-proliferative effect of prostacyclin analogues on lung fibroblasts. In IPF cells, the anti-fibrotic effect of prostacyclin has not been shown to correlate with the overall cAMP level, but rather with its accumulation in the nucleus [20]. This nucleus-specific signalling in IPF is indirectly supported by the observation that treprostinil activates the target of cAMP, Akt, only in the nucleus within 30 min, but has no effect on the compartmented cell localization of its overall expression.

The induction of the epithelial-to-mesenchymal transition of epithelial cells by cigarette smoke and the reduction of E-cadherin were prevented by the prostacyclin analogue Iloprost [36]. In human bronchial fibroblasts, the activation of the prostacyclin receptor significantly reduced TGF-β1-induced myo-fibroblast differentiation, proliferation, and extracellular matrix synthesis, as well as the secretion of interleukin-6 and PAI-1 (plasminogen-activator inhibitor) [37]. In line with these reports, treprostinil upregulated PTEN and p21(Waf1/Cip1), which have been reported to inhibit the proliferation of IPF cells and fibroblasts [21,23].

However, the contribution of mitochondria and autophagy to the pathogenesis and progress of IPF remains unclear. Some studies suggest that the effect of autophagy on IPF might depend on the stage of the disease [38,39]. In proliferating fibroblasts within the fibrotic tissues of mice, the expression of beclin1, LC3, and p62 were upregulated [39]. However, in human tissues, the expression of beclin1 and LC3 were reduced in IPF patients when compared to controls [35,40]. As described above, treprostinil downregulated p62, but increased the expression of beclin1, LC3 II, and parkin in IPF cells, indicating increased autophagy. In line with these data, reduced mitochondrial fission and ROS were correlated with low p62 levels and increased autophagy in neuronal cells [41]. However, it was also reported that neither the reduced mitochondria fission nor the reduced ROS affected the expression of beclin1 and LC3 II.

Treprostinil upregulated the expression of PTEN and downregulated the phosphorylation of mTOR [42]. A similar signalling cascade was reported in a model of pulmonary fibrosis where Akt controlled autophagy through the upregulation of PTEN [43].

In conclusion, the presented data suggest that treprostinil exerts part of its beneficial effect on lung fibrosis development by reducing mitochondrial fission and modulating autophagy, and might thereby reduce the development or progression of fibrosis in lung cells. Further investigations are needed to understand the contribution of mitochondrial fission and fusion to the pathogenesis of IPF, and whether prostacyclin analogues such as treprostinil can beneficially regulate mitochondrial activity and function.

## 4. Materials and Methods

### 4.1. Cells

Human lung fibroblasts were isolated from the non-diseased resected lung tissues of cancer patients with lung metastases (*n* = 6, 3 male, 3 female, age range: 45–72 years).

Fibroblast-like cells (spindle shape) were isolated from resected lung tissues obtained from IPF patients (*n* = 6, 4 male, 2 female, age range: 35–64 years).

Lung tissues were provided by the Department of Thoracic Surgery (University Hospital of Vienna, Austria) between 2015 and 2018. The protocol was approved by the ethical committee of the Medical University of Vienna (EK:1147/2015).

Fibrosis was diagnosed as category C, following the classifications of Eurotransplant [44] and ATS/ERS [45]. IPF was diagnosed by histology and radiology. Patients with autoimmune disorders or connective tissue diseases were excluded.

Fibroblasts were isolated using a selective medium (CnT-PR-F, CellnTec, Bern, Switzerland). Subsequent to passage 1, fibroblasts were propagated in RPMI-1640 supplemented with 10% foetal calf serum, 20 mM HEPES, 1 x amino acid mix, and 8 mM L-glutamax (all Gibco/BRL, Baar, Switzerland). Cell characterization was performed by immunochemistry as described earlier [20].

### 4.2. Drugs

Human recombinant TGF-β1 was purchased from R&D Systems (cat# 7754-BH, Abington, UK) and activated by acidification as described by the distributor.

Treprostinil was obtained from the United Therapeutics Corporation (Research Triangle Park, Silver Spring, NC, USA) and dissolved in PBS before being sterilized by filtration (20 μm filter, Milipore, Burlington, VT, USA). The concentration of the drug (1 × 10^−8^ M) has been shown to be effective on different cell biological aspects of fibrosis in clinical studies [20] and in previous in vitro studies [20,21,22,25,35,43].

2′,3′-Dideoxyadenosine (DDA) is a specific adenylyl cyclase inhibitor and, therefore, blocks the generation of cAMP, which we reported in earlier studies [21,22,25].

### 4.3. Western Blotting

Proteins were isolated with the RIPA buffer (radio-immune precipitation assay; cat# R0278, Sigma-Aldrich, Buchs, Switzerland) from the confluent cell layer before and after treatment with treprostinil. The protein content was determined using a BCA protein analysis kit (cat# 23227, Thermofisher Scientific, Reinach, Switzerland). Denatured proteins (20 µg) were size-fractionated through SDS-PAGE (2–12%, cat# M41212, GeneScript, Leiden, The Netherlands) before being transferred onto a nitrocellulose membrane (cat# 88018, Thermofisher Scientific). Target proteins were detected by primary antibodies (Table 1) by overnight incubation at 4 °C, followed by 3 washes with phosphate-buffered saline (PBS), and were then incubated with horseradish peroxidase (HRP)-labelled secondary antibodies (1 h at room temperature). Subsequent to 3 washes with PBS, the membranes were incubated with a light-emitting substrate and protein bands were visualized with the Azure C300 digital imaging system (Axonlab, Baden, Switzerland). The protein band intensity was analysed using ImageJ (ImageJ, Java v1.8.0_172, NIH, Bethesda, MD, USA) as described earlier [23].

### 4.4. Immunofluorescence Microscopy

Cells were seeded into 8-well slides (cat# 94.6140.802, Sarstedt) and allowed to adhere overnight. Following treatment under different conditions, the cells were fixed by incubation in 4% formaldehyde (in PBS, 2 × 15 min, at room temperature). The slides were washed 3 × with PBS containing 0.1% TWEEN20 before being incubated overnight (4 °C) with a primary antibody (Table 1). After 3 washes with PBS, the slides were incubated with an Alexa488-labelled secondary antibody (cat# 11001, Thermofisher) for 1 h at room temperature. Nuclei were stained with DAPI (4′,6-diamidino-2-phenylindole). Immunofluorescence microscopy was obtained using an Olympus microscope and analysed using the imaging software FIJI, ImageJ2 as described earlier [46].

### 4.5. Mitochondrial Phenotyping

Cells were seeded into 8-well slides, treated with various conditions, and fixed in 4% formaldehyde (in PBS, 2 × 15 min, at room temperature). Cell membranes were permeabilised by a 15 min incubation in PBS containing 0.1% Triton-X100, followed by 2 washes with PBS. Unspecific binding was inhibited by 5% bovine serum albumin in PBS + 0.1% TWEEN20 for 30 min at room temperature. The slides were incubated with primary antibodies to cytochrome C (Table 1) overnight (4 °C). The slides were washed with PBS before being incubated with secondary antibodies for 30 min (37 °C), and were then stained with DAPI (5 min). Immunofluorescence photographs were obtained using an A1R confocal laser scanning microscope (Nikon, Amsterdam, The Netherlands).

### 4.6. Statistics

All data are presented as means ± S.E.M. and were analysed using the software Prism9.

The null hypothesis: No difference under any condition. Statistical analysis: Student’s *t*-test for comparing control to treatment and Mann–Whitney U-test for difference between two conditions. A *p*-value < 0.05 was considered significant.

## Figures and Tables

**Table 1:** Antibodies used for Western blotting and immunofluorescence microscopy.

Protein Target	Source	Cat#	Dilution	Species
total Akt	CST ^1^	4691	1:2000	rabbit
phosphorylated Akt	CST	4060	1:2000	rabbit
alpha-tubulin	TFSci ^2^	32-2700	1:2000	mouse
beclin1	CST	3495	1:2000	rabbit
total CREB	CST	9197	1:1000	rabbit
phosphorylated CREB	CST	9198	1:1000	rabbit
cytochrome C	BD ^3^	556432	1:1000	rabbit
LC3 I/II	CST	12741	1:2000	rabbit
mitofusin1	Abcam ^4^	Ab221661	1:2000	rabbit
mitofusin2	Abcam	Ab124773	1:2000	rabbit
total mTOR	CST	2983	1:2000	rabbit
phosphorylated mTOR	CST	2974	1:2000	rabbit
MTCO2	Abcam	Ab79393	1:1000	rabbit
MTCO4	CST	4850	1:2000	rabbit
p21^(Waf1/Cip1)^	Abcam	188224	1:1000	rabbit
p62	R&D ^5^	MAB8028	1:1000	mouse
parkin	Abcam	Ab77924	1:2000	mouse
PINK1	Abcam	Ab300623	1:1000	rabbit
PTEN	CST	9188	1:2000	rabbit
anti-mouse HRP	SA ^6^	A9917	1:2000	goat
anti-mouse Alexa 488	TFSci	A21121	1:500	goat
anti-rabbit HRP	SA	A9169	1:2000	goat

^1^ CST: Cell Signalling Technology, Danvers, MA, USA; ^2^ TFSci: ThermoFisher Scientific, Waltham, MA, USA; ^3^ BD: Becton Dickinson Bioscience, Franklin Lakes, NJ, USA; ^4^ Abcam, Cambridge, UK; ^5^ RD: R&D Systems, Minneapolis, MN, USA; ^6^ SA: Sigma Aldrich, Burlington, MA, USA.

## Data Availability

The original data can be requested from the corresponding author, C.L.
